# High prevalence of *Plasmodium falciparum* gametocyte infections in school-age children using molecular detection: patterns and predictors of risk from a cross-sectional study in southern Malawi

**DOI:** 10.1186/s12936-016-1587-9

**Published:** 2016-11-04

**Authors:** Jenna E. Coalson, Jenny A. Walldorf, Lauren M. Cohee, Miriam D. Ismail, Don Mathanga, Regina Joice Cordy, Matthias Marti, Terrie E. Taylor, Karl B. Seydel, Miriam K. Laufer, Mark L. Wilson

**Affiliations:** 1 Department of Epidemiology, University of Michigan School of Public Health, Ann Arbor, MI USA; 2 Division of Malaria Research, Institute for Global Health, University of Maryland, Baltimore, MD USA; 3 Malaria Alert Centre, College of Medicine, University of Malawi, Blantyre, Malawi; 4 Department of Immunology and Infectious Disease, The Harvard School of Public Health, Boston, MA USA; 5 Department of Osteopathic Medical Specialties, College of Osteopathic Medicine, Michigan State University, East Lansing, MI USA

**Keywords:** Epidemiology, Malaria, Gametocytes, qRT-PCR, Molecular testing, Transmission reservoir, School-age children

## Abstract

**Background:**

In endemic areas, many people experience asymptomatic *Plasmodium* infections, particularly older children and adults, but their transmission contribution is unknown. Though not the exclusive determinant of infectiousness, transmission from humans to mosquitoes requires blood meals containing gametocytes. Gametocytes often occur at submicroscopic densities, challenging measurement in human populations. More sensitive molecular techniques allow better characterization of gametocyte epidemiologic patterns.

**Methods:**

Approximately 30 households were selected from each of eight sites in southern Malawi during two cross-sectional surveys. Blood was sampled from 623 people during the dry season and 896 the following rainy season. Among people PCR-positive for *Plasmodium falciparum*, mature gametocytes were detected by qRT-PCR. Regression models evaluated predictors of gametocyte carriage and density in the total population and among those with PCR-positive infections.

**Results:**

The prevalence of gametocyte carriage by molecular testing was 3.5% during the dry season and 8.6% during the rainy season, and by microscopy 0.8 and 3.3%, respectively. Nearly half of PCR-positive infections carried gametocytes, regardless of recent symptom status. Among *P. falciparum*-infected people, only living in unfinished houses and age were significantly associated with gametocyte presence. Infected people in unfinished houses had higher odds of carrying gametocytes (OR 2.24, 95% CI 1.16–4.31), and 31% (95% CI 3–65%) higher gametocyte density than those in finished houses. School-age children (5–15 years), had higher odds than adults (≥16 years) of having gametocytes when infected (OR 2.77, 95% CI 1.47–5.19), but 31% (95% CI 11–47%) lower gametocyte density. Children <5 years did not have significantly higher odds of gametocyte carriage or density when infected than adults.

**Conclusions:**

School-age children frequently carry gametocytes in communities of southern Malawi and represent an under-recognized reservoir of infection. Malaria elimination strategies should address these frequently asymptomatic reservoirs, especially in highly endemic areas. Improved household construction may also reduce the infectious reservoir.

**Electronic supplementary material:**

The online version of this article (doi:10.1186/s12936-016-1587-9) contains supplementary material, which is available to authorized users.

## Background

Concerted international efforts have made strides toward malaria control and elimination [1, 2], but the persistence of the disease in some settings suggests that key sources of transmission may be overlooked by current intervention strategies. Recent studies have brought attention to the high burden of asymptomatic and submicroscopic *Plasmodium* infections [3–8], which are widespread in areas where malaria is endemic, but the extent of infectiousness and contribution to transmission dynamics of these people is not fully understood [9]. Transmission from an infected human to a mosquito requires that at least one male and one female gametocyte, the sexual stages of *Plasmodium* parasites, are ingested in a blood meal [10], but few studies have characterized community-wide distribution of gametocyte carriers to identify which groups of people in endemic areas are potentially infectious. Improved understanding of gametocyte carriage may illuminate gaps in intervention strategies that have enabled transmission to persist, and aid public health workers in identifying targets to more effectively interrupt transmission.

Many previous studies have suggested that the odds that a *Plasmodium falciparum* infection carries gametocytes decrease with age, but typically relied on microscopy to detect gametocytes [11–28]. The lower limit of detection of microscopy is approximately 40 parasites/µL, depending on slide preparation, the skill of the reader, and the number of fields viewed [29], but gametocytes tend to occur at low densities (<5% of total parasite burden), and gametocyte densities of <1/μL can infect some mosquitoes [10, 30]. Modern molecular testing techniques that detect gametocyte-specific mRNA transcripts, such as quantitative reverse transcription polymerase chain reaction (qRT-PCR) and quantitative nucleic acid sequence based amplification (QT-NASBA), offer greatly improved sensitivity over that of microscopy, potentially down to the level of <1 parasite/µL [10, 31, 32]. Thus, the poor sensitivity of microscopy limits inferences about gametocyte carriage patterns, making molecular-based results critical to understanding human transmission reservoirs. Molecular gametocyte detection methods have become increasingly common in the past 15 years, though typically they have been used in clinical trials among symptomatic populations and/or exclusively in young children [33–40]. Only a few studies have used molecular methods to describe population gametocyte epidemiology [21, 25, 27, 28, 41]. Previous research from southern Malawi found that school-age children have the highest prevalence of infection [42], but their gametocyte carriage and contribution to transmission remains unclear.

To accurately define the gametocyte reservoirs in a high burden setting, we used specimens collected from two cross-sectional surveys in Malawi. Community-based studies of people were undertaken during two seasons and in households from areas that experienced different transmission intensities. The study hypothesis was that the odds of being gametocytaemic when infected would decrease with age, but that this association would be weaker in sites with lower transmission intensity, where less frequent exposures might delay the development of immune responses to gametocytes. This study is the first to explore the epidemiology of gametocyte carriage among people living in communities of southern Malawi using sensitive molecular tests.

## Methods

### Ethics, consent, and permissions

The study was carried out under the auspices of the Malawi International Center of Excellence for Malaria Research (ICEMR), and all methods were approved by the independent Institutional Review Boards (IRBs) of the University of Malawi College of Medicine, the University of Maryland, Baltimore, and Michigan State University. The University of Michigan IRB deemed their investigators’ role in the project to be ‘not regulated’ given existing approvals. Informed consent was obtained from all participants or their guardians, as appropriate. Assent was also obtained from participants 13–17 years old.

### Study setting

Malaria is endemic throughout Malawi, with seasonal variation driven by the annual rainy season that runs from November/December through March/April [43]. The majority of infections are attributable to *P. falciparum*, though both *Plasmodium ovale* and *Plasmodium malariae* have been detected, frequently as mixed infections with *P. falciparum* [44]. Vector information is somewhat limited, but *Anopheles arabiensis*, *Anopheles gambiae* sensu stricto, and *Anopheles funestus* have all been identified [43]. There is geographic heterogeneity, with risks being highest in humid lowland regions and in rural areas [43, 45]. This study reports on sites from three districts: Blantyre, Thyolo, and Chikhwawa. Blantyre is a large urban area in the highlands, expected to have relatively low transmission; Thyolo is a rural area located mostly in the highlands, expected to have moderate transmission; and Chikhwawa is a rural area found in the hot, low-lying Shire Valley, and is known to have relatively high parasite transmission, with an estimated EIR of 172 infectious bites per year [43].

### Study design

**Fig. 1:**
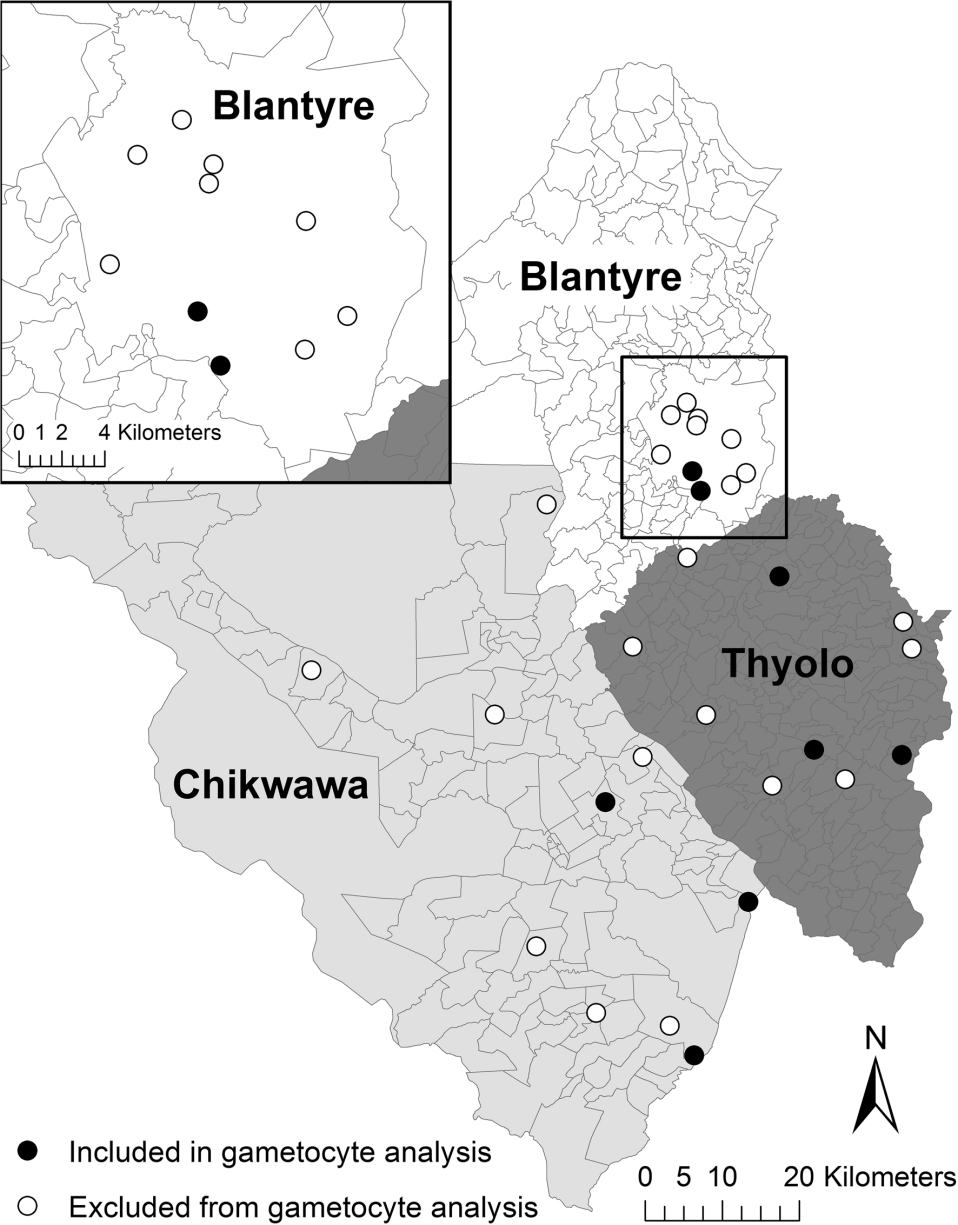
Enumeration areas (EAs) in the ICEMR-Malawi cross-sectional study

Data were collected in three districts of southern Malawi during cross-sectional surveys at the end of the dry season (September–October) 2012 and rainy season (April–May) 2013. A group of ~30 households was chosen within each of 10 enumeration areas (EAs) in each of the three districts. These 30 EAs were randomly selected using two-stage cluster sampling as described by Walldorf et al. [42]. The present study involved a subset of eight EAs that were chosen to include the diverse environmental and epidemiological characteristics of the three districts (Fig. 1), while considering supply availability and transport schedule during the dry season 2012. The survey team returned to the same eight sites for the rainy season 2013 that were sampled during the dry season 2012. Communication with local health representatives and chiefs encouraged high levels of participation, but households and individuals were not specifically linked between the two surveys.

At each visit, the field team interviewed household members in the local language (Chichewa) to obtain information about household-level variables and individual-level variables for all members of the household (defined as anyone who slept in the household for at least 2 weeks during the previous month). A standardized questionnaire sought data on household demographics (i.e. age, sex, relationship of all members), house construction, use of malaria interventions, self-reported recent symptoms of disease, and recent treatment-seeking behaviours.

### Sample collection and preservation

Nurses working with each field team measured axillary body temperature and took peripheral blood samples from all subjects ≥6 months old (mo) who were present and consented (assented, if appropriate) to participate in the study. The nurses collected drops of blood onto slides as thick smears for microscopy and onto filter paper for DNA isolation and PCR. To preserve RNA, whole blood samples of ~50 μL were collected into 250 μL of RNAprotect^®^ (Qiagen Inc., Valencia, CA). RNA samples were collected from the first ~50 to 80 available and consented subjects ≥6 months of age from each site during the first dry season of RNA sampling, and from all available and consented individuals during the subsequent rainy season. The presence of *P. falciparum* infection and gametocytes was determined by microscopy and molecular methods in all sampled subjects of the eight EAs from whom RNA samples were collected during the dry season 2012, and the same eight EAs during the subsequent rainy season 2013 (Fig. 1).

### Microscopy procedures

Thick smears were air dried, methanol fixed, and Giemsa-stained upon delivery to the laboratory at the end of each day of sample collection. Thick smears were read at 100× objective magnification by two trained microscopists, who independently recorded the number of sexual and asexual parasites seen per 200 white blood cells (WBC). The reads were considered discrepant if one reader recorded presence of asexual *Plasmodium* parasites and the other did not, if one reader counted more than ten times as many asexual parasites as the other when the lowest reader counted fewer than 20, or if one reader counted more than twice as many as the other when both counted 20 or more. Discrepant slides were sent to a third reader. All readers were blinded to the results recorded by the other readers. The final smear value was recorded as zero if two readers reported the slide negative for asexual parasites. Using an assumed WBC count of 8000/µL, the final asexual smear value per µL of positive slides was estimated using the geometric mean of the two closest reads multiplied by 40. Since low gametocyte densities are known to be common, samples were considered microscopy-positive for gametocytes when any reader counted at least one gametocyte.

### Molecular testing

All dried blood spots underwent quantitative PCR to detect *P. falciparum* infection by the presence of the lactate dehydrogenase gene, as described previously [42]. qRT-PCR was conducted on the preserved RNA of all PCR-positive and ~1% of PCR-negative individuals to test for stage-specific *P. falciparum* mRNA. qRT-PCR used a multiplex assay developed by Joice et al. [46] and validated in a clinical trial in Uganda by Chang et al. [47] that distinguishes mature (stage IV–V) gametocytes from other stages.

Preserved RNA samples were stored at −80 °C until thawed for RNA extraction and testing. After thawing, samples were spun in a microfuge at room temperature to obtain a pellet. RNA was extracted from the selected samples using RNeasy Plus Mini-Kits^®^ (Qiagen Inc., Valencia, CA) and treated with RNase-free DNase Sets^®^ (Qiagen Inc., Valencia, CA) to eliminate parasite gDNA. Reverse transcription was performed using Superscript complementary DNA (cDNA) Synthesis Kits^®^ (Life Technologies™, Carlsbad, CA) in a double reaction compared to the standard protocol, using 50 ng/µL random hexamers as the primer. If PCR could not be performed immediately, the resulting cDNA samples were stored overnight at −20 °C.

Primer/probe mixes were created for qRT-PCR by combining 18 µL of forward primer and 18 µL of reverse primer at a concentration of 900 nM, 5 µL of probe at a concentration of 250 nM, and 59 µL of nuclease-free water. These mixes were stored in dark boxes at −20 °C. Each well of the qRT-PCR reaction consisted of 10 µL of ABI TaqMan Gene Expression Master Mix^®^ (Life Technologies™, Carlsbad, CA), 1 µL of the primer/probe mix, 5 µL of nuclease-free water, and 4 µL of cDNA sample. The assay was performed in triplicate for each stage-specific marker. Mature gametocyte detection involved the gene *PF14_0367*. The results of the qRT-PCR were recorded as positive for gametocytes if at least two of three wells had a clear amplification curve.

The density of circulating gametocytes was estimated using the mean threshold cycle (Ct) values of the two or three positive wells from qRT-PCR [47]. Briefly, the relationship between Ct values and gametocyte density was estimated using linear regression of samples that had microscopically-quantified gametocyte densities. The regression results were then used to infer the densities from the Ct values for samples that were not identified as gametocyte-positive by microscopy.

### Data management and analysis

All data were stored in a Research Electronic Data Capture (REDCap) system (Vanderbilt University) hosted at the University of Malawi College of Medicine [48]. New variable creation and all analyses were performed in Statistical Analysis System (SAS) version 9.4 (SAS Institute, Cary, NC).

Because we observed within-district heterogeneity in EA-level parasite prevalence and because the EA site selection was limited by supply arrival and scheduling, hence not random, selected EAs were not considered representative of their districts. Thus, district was not a valid proxy for local transmission intensity, and was not included in statistical analysis. Instead the relative *P. falciparum* transmission intensity was approximated for each site using PCR-based prevalence data from a previous rainy season survey undertaken by ICEMR Malawi (unpublished data). The 30 study sites were classified into tertiles by this baseline *P. falciparum* prevalence in order to make relative comparisons across EAs. The lowest tertile included sites with PCR-based prevalence of 0 to <8%, the middle tertile 8% to <15%, and the highest tertile ≥15%. Testing for gametocytes was performed on two sites in the lowest tertile, four in the middle tertile, and two in the highest tertile.

Socioeconomic status (SES) variables were collected at the household level. Ten variables pertaining to SES (ownership of several assets, having a regular source of income, frequency of food shortages, and education) were combined into a single SES indicator variable based on the wealth index method of Filmer and Pritchett [49]. Principal Component Analysis (PCA) was performed in SAS using *proc factor* to obtain a weight for each indicator. This was used to evaluate their relative contribution to the total household score by survey and summed to obtain an index score. The PCA-weighted SES index score was applied to all individuals within that household. The individuals from all 30 EAs from a given survey were grouped into quartiles by the SES index score. The subset of eight sites where RNA was sampled was extracted from the quartiles assigned to the entire dataset for a given survey, leading to uneven sample sizes for each quartile within this analysis.

House construction was assessed separately from the overall SES index, as it may be more directly associated with *Plasmodium* transmission if poor design or construction increase exposure to *Anopheles* vectors. The roof, floors, and walls of each house were classified as natural, rudimentary, or finished. Each house was categorized as ‘finished’ overall if at least two of the roof, walls, and floors were classified as finished rather than natural or rudimentary, and ‘unfinished’ if none or only one of the three was finished. The eaves were separately categorized as ‘open’ or ‘closed’ in order to assess whether they had an independent impact after considering other household construction variables.

Age groups were initially defined in five categories for descriptive results, using the categories 6 months ≤5 years, 5–10 years, >10–15 years, 16–30 years, and >30 years, but sample size constraints led us to collapse these categories for the majority of the analyses into young children (6 mo ≤5 year), school-age children (5–15 year), or adults (≥16 year). Bed net availability and use were determined by whether each household owned at least one bed net, and which members of the household reportedly slept under a bed net on the previous night. Individuals were classified as living in a household (1) without access to any nets, (2) with at least one net but not using it the previous night, or (3) with at least one net and sleeping under it the previous night.

Symptoms of malaria were defined as reported history of any fever in the previous 2 weeks or temperature ≥37.5 °C at time of survey. Data also were collected on the use of any treatments in the previous 2 weeks, including all anti-malarial medications.

Gametocyte carriers were defined as people who tested positive for any *P. falciparum* gDNA by PCR and subsequently tested positive for the mature gametocyte marker by qRT-PCR. Univariate analyses of potential predictors of gametocyte infection were performed using Chi squared tests of association, or Fisher’s exact tests for variables with expected values of five or fewer. Continuous variables were analysed by *t* test when normal and Wilcoxon-Mann–Whitney tests when non-normal. Logistic regression models were used to evaluate the potential predictors of gametocyte carriage in the total study population, and among the subpopulation that was parasitaemic by PCR, in the context of multivariable adjustments. Multilevel models, using the *proc glimmix* command, were analysed to account for clustering at the household and EA levels and prevent overestimation of statistical significance. As the estimated gametocyte densities were overdispersed, negative binomial and zero-inflated negative binomial regression models were tested in order to evaluate factors associated with estimated gametocyte density among people with PCR-positive infections.

## Results

**Fig. 2:**
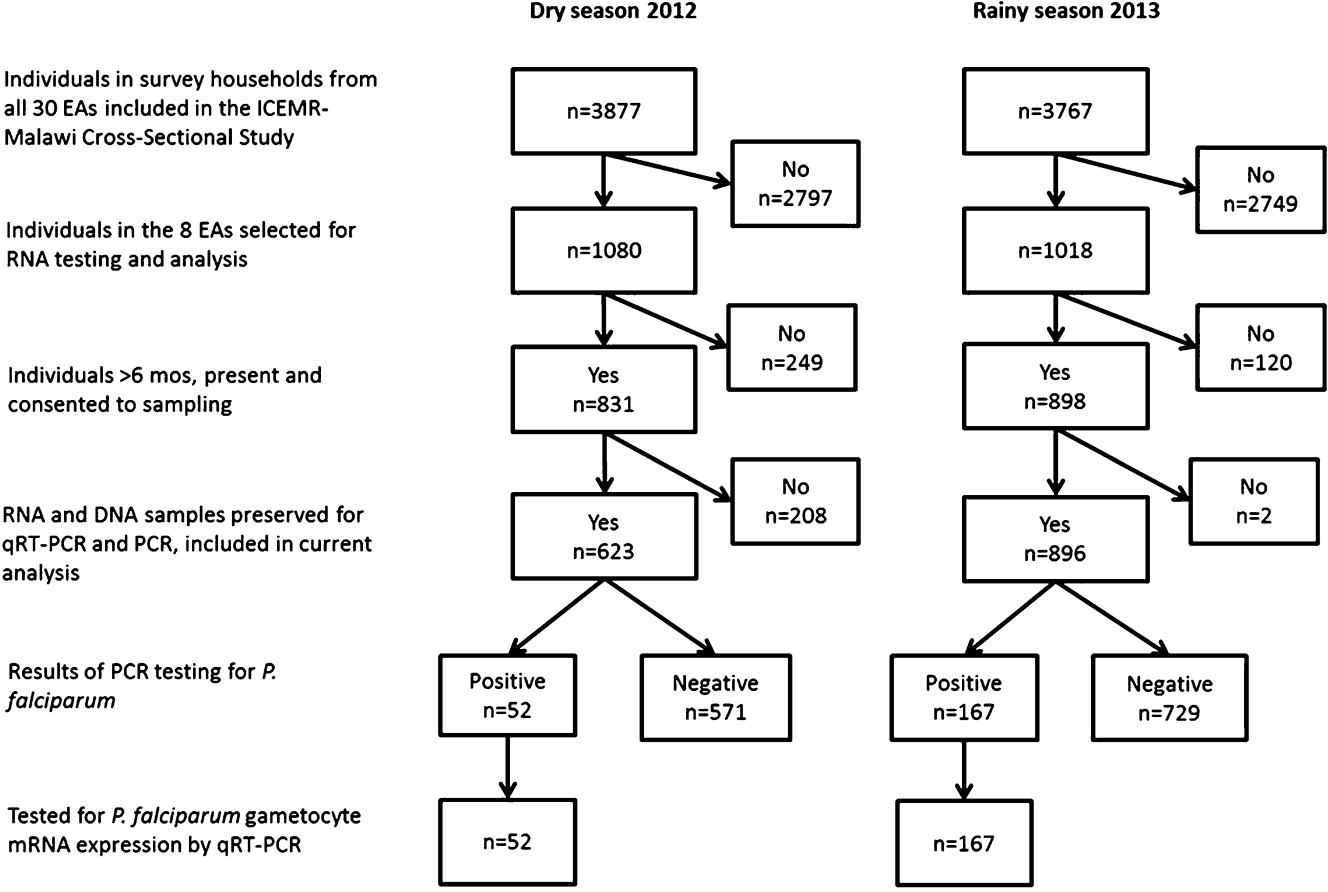
Sampling diagram for molecular testing of gametocytes by survey

**Fig. 3:**
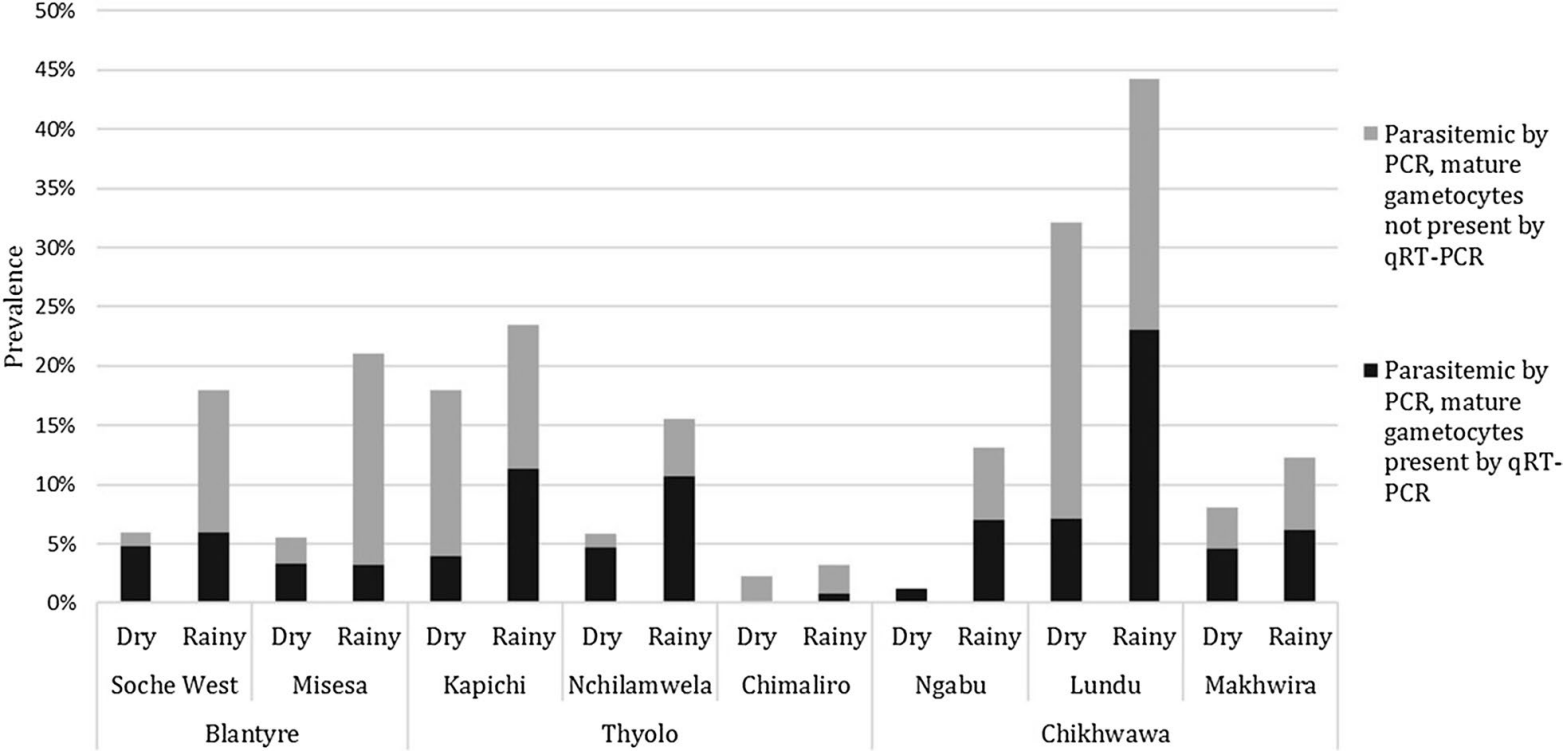
Prevalence of parasites and gametocytes by enumeration area and season

Overall, 623 individuals from 214 households from the dry season and 896 individuals from 248 households from the rainy season formed the study population for molecular analysis (Fig. 2; Table 1). Individuals who provided samples were significantly more likely to be female, children under 5 years of age, and to have had a recent fever than those that were not sampled in both surveys. A total of 52 (8.3%) samples from the dry season and 167 (18.6%) from the rainy season tested positive for *P. falciparum* parasite gDNA by PCR and were subsequently tested by qRT-PCR for gametocytes. The prevalence of mature gametocytes, like the prevalence of infection overall, was generally higher during the rainy season 2013 than the dry season 2012 in each EA, but the proportions of infections that contained gametocytes by season was variable (Fig. 3).

**Table 1:** Characteristics of the study population and prevalence of parasitemia and gametocytemia by molecular testing

	n	*P. falciparum* parasitemia^a^		Mature gametocytemia	
		n (%)	p value	n (%)	p value
Total	1519	219 (14.4%)		99 (6.5%)	
*Microscopy results*					
Positive for any parasitemia	127 (8.5%)	108 (85.0%)		65 (51.2%)	
Negative for any parasitemia	1373 (91.5%)	107 (7.8%)	<*0.0001*	34 (2.5%)	<*0.0001*
*Season*					
Dry 2012	623 (41.0%)	52 (8.4%)		22 (3.5%)	
Rainy 2013	896 (59.0%)	167 (18.6%)	<*0.0001*	77 (8.6%)	<*0.0001*
*EA transmission intensity* ^*b*^					
Low	408 (26.9%)	22 (5.4%)		10 (2.5%)	
Medium	777 (51.2%)	93 (12.0%)		44 (5.7%)	
High	334 (22.0%)	104 (31.1%)	<*0.0001*	45 (13.5%)	<*0.0001*
*Sex*					
Male	585 (38.6%)	91 (15.6%)		44 (7.5%)	
Female	931 (61.4%)	128 (13.8%)	0.33	55 (5.9%)	0.22
*Age (mean 17.3* *years old)*					
Young children, 6 months to <5 years old	290 (19.1%)	21 (7.2%)		9 (3.1%)	
School-age children, 5–15 years old	564 (37.2%)	127 (22.5%)		68 (12.1%)	
Adults, ≥16 years old	662 (43.7%)	71 (10.7%)	<*0.0001*	22 (3.3%)	<*0.0001*
*SES quartile* ^*c*^					
Lowest	397 (26.2%)	60 (15.1%)		35 (8.8%)	
2nd	427 (28.2%)	74 (17.3%)		29 (6.8%)	
3rd	437 (28.9%)	67 (15.3%)		29 (6.7%)	
Highest	252 (16.7%)	18 (7.1%)	0.01	6 (2.4%)	<0.01
*Household construction*					
Unfinished	816 (53.7%)	143 (17.5%)		71 (8.7%)	
Finished	703 (46.3%)	76 (10.8%)	<*0.001*	28 (4.0%)	<*0.001*
*Eaves*					
Open	390 (25.7%)	69 (17.7%)		34 (8.7%)	
Closed	1129 (74.3%)	150 (13.3%)	0.03	65 (5.8%)	0.04
*Bednet use*					
Slept under a net previous night	979 (64.5%)	137 (14.0%)		66 (6.7%)	
Net available but not used	364 (24.0%)	67 (18.4%)		28 (7.7%)	
No nets in household	176 (11.6%)	15 (8.5%)	<0.01	5 (2.8%)	0.09
*IRS in previous 12* *months*					
Yes	234 (15.5%)	31 (13.3%)		17 (7.3%)	
No	1276 (84.5%)	185 (14.5%)	0.62	82 (6.4%)	0.63
*Fever in the previous 2* *weeks*					
Yes	266 (17.6%)	36 (13.5%)		18 (6.8%)	
No	1249 (82.4%)	183 (14.7%)	0.64	81 (6.5%)	0.87
*Fever in past 2* *weeks, any fever, headache, or rigors/chills in past 48* *h, or temperature ≥37* *°C*					
Yes	442 (29.2%)	58 (13.1%)		29 (6.6%)	
No	1073 (70.8%)	161 (15.0%)	0.34	70 (6.5%)	0.98
*Any treatment sought in the previous 2* *weeks*					
Yes	301 (19.9%)	36 (12.0%)		18 (6.0%)	
No	1215 (80.1%)	183 (15.1%)	0.17	81 (6.7%)	0.67
*Antimalarial taken in the previous 2* *weeks*					
LA	56 (3.7%)	5 (8.9%)		2 (3.6%)	
Other antimalarial^d^	10 (0.7%)	3 (30.0%)		1 (10.0%)	
None	1453 (95.7%)	211 (14.5%)	0.17	96 (6.6%)	0.47

*EA* Enumeration area; *ICEMR* International Center of Excellence for Malaria Research; *IRS* indoor residual spraying; *LA* lumefantrine artemether; *SES* socioeconomic status

Italized p values are significant at level 0.05 after Holm-Bonferroni adjustment for multiple comparisons

^a^Parasitemia as detected by PCR for *P. falciparum* lactate dehydrogenase (LDH)

^b^Tertiles of parasite prevalence established for all 30 EAs from the first survey (rainy season 2012) were used as a proxy estimate of transmission intensity in the EA. See "Methods" section for details

^c^Mantel-Haenszel p value was used for trends with SES; all others were Pearson Chi square test p values

^d^‘Other’ antimalarials included chloroquine, quinine, or sulfadoxine-pyrimethamine

### Prevalence of gametocytes in the community

The prevalence of gametocyte carriage detected by the molecular testing strategy was 6.5% in the total study population of 1519 subjects. The estimated prevalence of gametocyte carriage was 3.5% (22 of 623) during the dry season and 8.6% (77 of 896) during the rainy season. These represent minimum estimates, as they exclude potential gametocyte carriers among individuals whose total parasite burden was under the LOD of PCR in the ICEMR Malawi laboratory (~2.7/μL), although no *P. falciparum* mRNA was detected in the ~1% of PCR-negative samples that were tested by qRT-PCR. In contrast, microscopy identified prevalences of only 0.8% (5 of 604) during the dry season and 3.3% (30 of 896) during the rainy season. An additional file compares the results of gametocyte detection by microscopy vs. qRT-PCR (Additional file 1).

Among the 219 individuals who were PCR-positive for *P. falciparum* infections, 99 (45.0%) tested positive for mature gametocytes by qRT-PCR. The distribution of individuals carrying PCR-positive infections and mature gametocytes by various characteristics of the study population is presented in Table 1. Of the 99 individuals with qRT-PCR-detected gametocytes, only 65 (65.7%) had patent infections. The remaining ~1/3 of gametocyte carriers had submicroscopic/subpatent infections.

### Predictors of gametocyte carriage in the study population

**Fig. 4:**
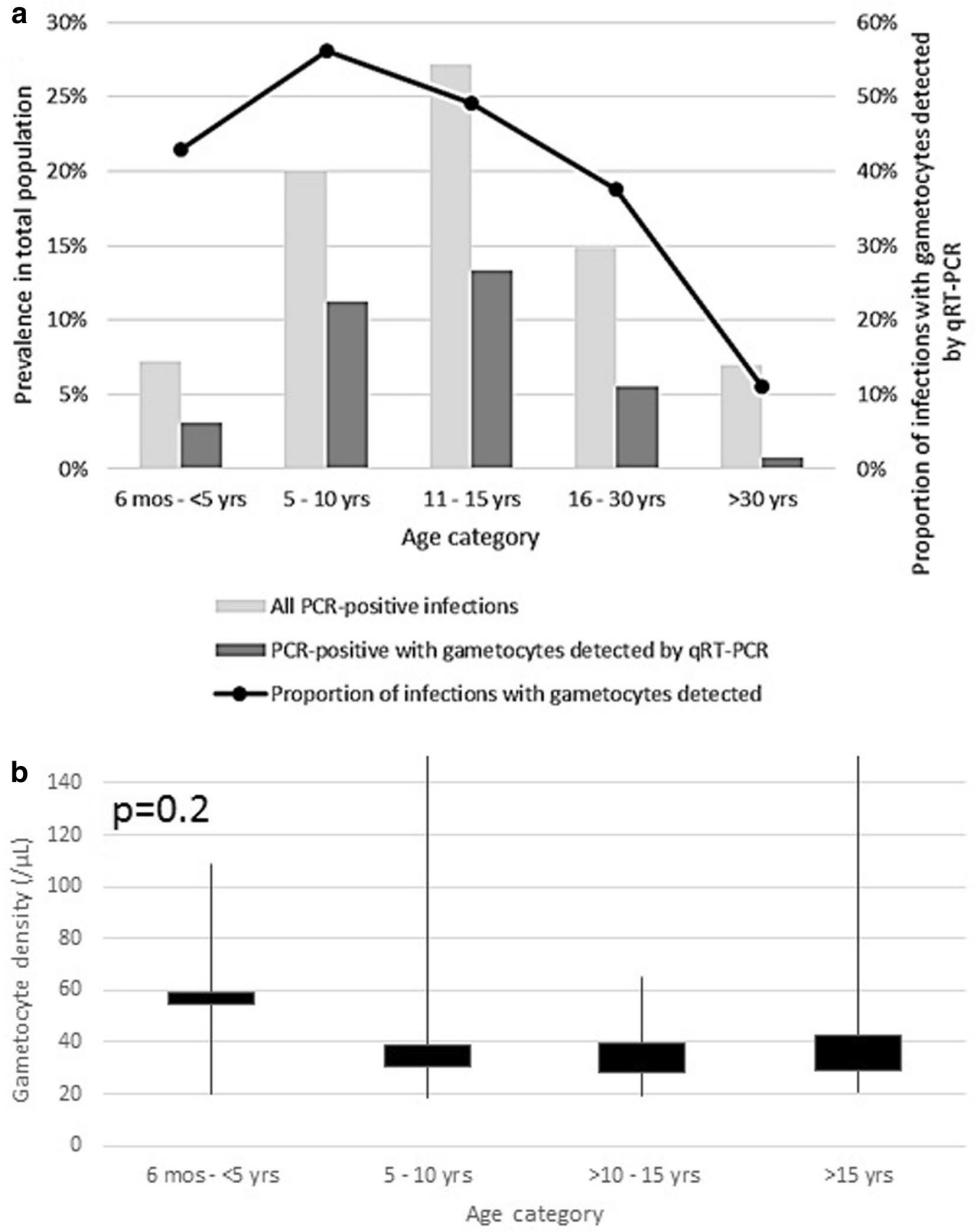
Parasite and gametocyte carriage by age. **a** The *bars* correspond to the left axis and present the prevalence of *P. falciparum* parasitaemia as detected by qPCR, as well as the prevalence of gametocyte carriers identified among them, by age category. The *line* corresponds to the right axis and displays the proportion of PCR-positive infections in which mature gametocytes were detected by qRT-PCR. **b** The distribution of Ct-based estimated gametocyte densities by age among those in whom mature gametocytes were detected by qRT-PCR. Kruskall-Wallis p value for any association with age, p = 0.20

To characterize gametocyte reservoirs for the purposes of targeting interventions, the strength of various predictors of gametocyte carriage were assessed in the community at large. These largely mirrored the predictors of overall PCR-detected parasitaemia, with age category being one of the strongest predictors of both parasitaemia and gametocyte carriage (p < 0.0001 for each) in univariate analysis. The prevalence of mature gametocytes in school-age children (12.1%) was higher than in young children (3.1%) or adults (3.3%). More specific detail on the age distribution of infections is presented in Fig. 4. Both the prevalence of infections containing gametocytes overall and the proportion of infections that contained gametocytes peaked among school-age children, and declined sharply for adults >30 years of age. Gametocytes were detected in only two of 259 (0.77%) people older than 30—only 11.1% of the 18 PCR-positive infections in this age group. The median estimated density of gametocytes was highest among children <5 years of age, but there was considerable variability, and the association with age was not statistically significant (Kruskall-Wallis p value = 0.20).

Notably, people who reported recent malaria symptoms were equally as likely to carry gametocytes as those that were asymptomatic (p = 0.87 for any fever in the previous 2 weeks). The prevalences of parasites and gametocytes were lower in individuals from households that did not have any bed nets compared to individuals from households that did have bed nets, whether or not the individuals reported using a bed net the previous night. Among households that owned any bed nets, the prevalences of parasites and gametocytes were higher among individuals who reported not sleeping under a net on the previous night (p < 0.01 and p = 0.09, respectively).

Multilevel logistic regression models were used to explore the predictors of gametocyte carriage in the total population (Table 2). Random intercepts at the household- and EA-levels were included to account for potential clustering within the data. Season, EA transmission intensity, and age category all significantly improved the model fit and were included in adjusted models to test all other potential predictors. Tests of interaction could not be included in the final model as small sample sizes led to instability of the estimates.

**Table 2:** Predictors of gametocyte carriage in the cross-sectional study population based on multilevel logistic regression

	Unadjusted POR (95% CI)	Adjusted POR (95% CI)^a^	Adjusted POR, multilevel model (95% CI)^a^
*Season*			
Dry, 2012	1.00 (ref)	1.00 (ref)	1.00 (ref)
Rainy, 2013	*2.57 (1.58*–*4.17)*	*2.31 (1.40*–*3.80)*	*2.23 (1.33*–*3.74)*
*EA transmission intensity* ^*b*^			
Low	1.00 (ref)	1.00 (ref)	1.00 (ref)
Medium	*2.39 (1.19*–*4.80)*	*2.66 (1.31*–*5.42)*	2.86 (0.73–11.25)
High	*6.20 (3.07*–*12.50)*	*5.03 (2.45*–*10.33)*	*5.42 (1.11*–*26.45)*
*Age*			
Young children, 6 months to <5 years old	0.93 (0.42–2.05)	0.87 (0.39–1.94)	0.84 (0.38–1.87)
School-age children, 5–15 years old	*3.99 (2.43*–*6.54)*	*4.01 (2.42*–*6.65)*	*4.10 (2.46*–*6.83)*
Adults, ≥16 years old	1.00 (ref)	1.00 (ref)	1.00 (ref)
*Household construction*			
Finished	1.00 (ref)	1.00 (ref)	1.00 (ref)
Unfinished	*2.30 (1.47*–*3.60)*	*2.10 (1.29*–*3.45)*	*1.87 (1.08*–*3.26)*
*Bednet use*			
Slept under net previous night	2.47 (0.98–6.22)	2.17 (0.84–5.61)	
Net available but not used	2.85 (1.08–7.51)	2.13 (0.79–5.80)	
No nets in household	1.00 (ref)	1.00 (ref)	
*SES quartile*			
Lowest	*3.96 (1.64*–*9.57)*	2.01 (0.78–5.19)	
2nd	*2.99 (1.22*–*7.30)*	1.84 (0.70–4.80)	
3rd	*2.91 (1.19*–*7.12)*	2.13 (0.85–5.36)	
Highest	1.00 (ref)	1.00 (ref)	
*Eaves*			
Closed	1.00 (ref)	1.00 (ref)	
Open	1.56 (1.02–2.41)	1.21 (0.74–1.97)	
*Fever in previous 2* *weeks*			
Yes	1.05 (0.62–1.78)	1.56 (0.89–2.74)	
No	1.00 (ref)	1.00 (ref)	
*Any antimalarial taken in previous 2 weeks* ^*c*^			
Yes	0.67 (0.21–2.17)	0.79 (0.23–2.65)	
No	1.00 (ref)	1.00 (ref)	
*IRS in previous year*			
Yes	1.14 (0.66–1.96)	1.34 (0.71–2.52)	
No	1.00 (ref)	1.00 (ref)	
*Sex*			
Male	1.30 (0.86–1.95)	1.09 (0.70–1.67)	
Female	1.00 (ref)	1.00 (ref)	

*CI* confidence interval; *EA* Enumeration area; *IRS* Indoor residual spraying; *N/A* not applicable; *POR* prevalence odds ratio; *SES* socioeconomic status

Italized values are significant at level 0.05 (with Holm-Bonferroni adjustment for multiple comparisons in crude PORs)

^a^Adjusted for season, EA transmission intensity, age category, and household characteristics (finished vs. unfinished)

^b^Tertiles of parasite prevalence established for all 30 EAs from the first survey (rainy season 2012) data were used as a proxy estimate of transmission intensity in the EA. See "Methods" section for details

^c^Antimalarials included were lumefantrine artemether, chloroquine, quinine, or sulfadoxine–pyrimethamine

Adjustment for the predictors in the final model generally led to attenuation of the prevalence odd ratios (PORs) compared to the estimates from unadjusted models, but most of the key predictors identified from univariate analysis remained statistically significant. The best predictive model included season, EA transmission intensity, age category, and household characteristics. The odds of gametocytes among school-age children was particularly notable, with the odds of prevalent gametocyte infection being 4.10 (95% confidence interval (CI): 2.46–6.83) times the odds in adults ≥16 years of age and 4.89 (95% CI 2.35–10.17) times the odds in young children 6 months <5 years of age, even after adjusting for season, EA transmission intensity, and household construction quality. Young children had slightly lower odds of having gametocytes than adults (POR 0.84, 95% CI 0.38–1.87), though the difference was not statistically significant.

### Predictors of gametocyte carriage among PCR-positive infected individuals

The odds of carrying gametocytes when infected were also investigated by testing associations only among the population of 219 PCR-positive individuals (Table 3). Gametocytes were more frequently detected by qRT-PCR in individuals who had patent infections (65 of 108, 60.2%) than those with PCR-positive, submicroscopic infections (34 of 107, 31.8%) (p < 0.0001). However, estimated asexual parasite density was not associated with odds of gametocyte presence (POR for a change of 1000 parasites/µL = 1.01, 95% CI 0.99–1.03).

**Table 3:** Predictors of gametocyte carriage among individuals PCR-positive for *P. falciparum,* n = 219

	n	Gametocytemic n (%)	Unadjusted POR (95% CI)	Adjusted POR (95% CI)^a^
*Season*				
Dry, 2012	52	22 (42.3%)	1.00 (ref)	1.00 (ref)
Rainy, 2013	167	77 (46.1%)	1.17 (0.62–2.19)	1.35 (0.70–2.62)
*EA transmission intensity* ^*b*^				
Low	22	10 (45.5%)	1.00 (ref)	1.00 (ref)
Medium	93	44 (47.3%)	1.08 (0.42–2.74)	1.13 (0.42–3.00)
High	104	45 (43.3%)	0.92 (0.36–2.31)	0.69 (0.26–1.85)
*Age*				
Young children, 6 months to <5 years old	21	9 (42.9%)	1.67 (0.61–4.54)	1.72 (0.62–4.79)
School-age children, 5–15 years old	127	68 (53.5%)	*2.57 (1.39*–*4.73)*	*2.77 (1.47*–*5.19)*
Adults, ≥16 years old	71	22 (31.0%)	1.00 (ref)	1.00 (ref)
*Household construction*				
Finished	76	28 (36.8%)	1.00 (ref)	1.00 (ref)
Unfinished	143	71 (49.7%)	1.69 (0.96–2.99)	*2.24 (1.16*–*4.31)*
*Bednet use*				
Slept under a net previous night	137	66 (48.2%)	1.86 (0.60–5.72)	2.12 (0.65–6.95)
Net available but not used	67	28 (41.8%)	1.44 (0.44–4.66)	1.30 (0.38–4.52)
No nets in household	15	5 (33.3%)	1.00 (ref)	1.00 (ref)
*SES quartile*				
Lowest	60	35 (58.3%)	2.80 (0.93–8.46)	2.18 (0.64–7.40)
2nd	74	29 (39.2%)	1.29 (0.44–3.82)	0.99 (0.29–3.35)
3rd	67	29 (43.3%)	1.53 (0.51–4.55)	1.30 (0.41–4.16)
Highest	18	6 (33.3%)	1.00 (ref)	1.00 (ref)
*Eaves*				
Closed	150	65 (43.3%)	1.00 (ref)	1.00 (ref)
Open	69	34 (49.3%)	1.27 (0.72–2.25)	1.24 (0.64–2.43)
*Fever in previous 2* *weeks*				
Yes	36	18 (50.0%)	1.26 (0.62–2.58)	1.35 (0.63–2.86)
No	183	81 (44.3%)	1.00 (ref)	1.00 (ref)
*Any antimalarial taken in previous 2 weeks* ^*c*^				
Yes	8	3 (37.5%)	0.72 (0.17–3.09)	0.54 (0.12–2.38)
No	211	96 (45.5%)	1.00 (ref)	1.00 (ref)
*IRS in previous year*				
Yes	31	17 (54.8%)	1.53 (0.71–3.28)	1.49 (0.54–4.10)
No	185	82 (44.3%)	1.00 (ref)	1.00 (ref)
*Sex*				
Male	91	44 (48.4%)	1.24 (0.72–2.13)	1.15 (0.66–2.03)
Female	128	55 (43.0%)	1.00 (ref)	1.00 (ref)
Parasite density^d^	215	N/A	1.01 (0.99–1.03)	1.01 (0.99–1.03)

CI confidence interval; IRS indoor residual spraying; PCR polymerase chain reaction; POR Prevalence odds ratio; SES Socioeconomic status

Italized values are those where the 95% CI does not contain 1.0

^a^Adjusted for season, EA transmission intensity, age category, and household characteristics (finished vs. unfinished)

^b^Tertiles of parasite prevalence established for all 30 EAs from the first survey (rainy season 2012) data were used as a proxy estimate of transmission intensity in the EA. See "Methods" section for details

^c^Antimalarials included were lumefantrine-artemether, chloroquine, quinine, or sulfadoxine-pyrimethamine

^d^Parasite density was estimated by microscopy. The POR was reported per 1000 parasite/µL increase in estimated parasitemia

**Fig. 5:**
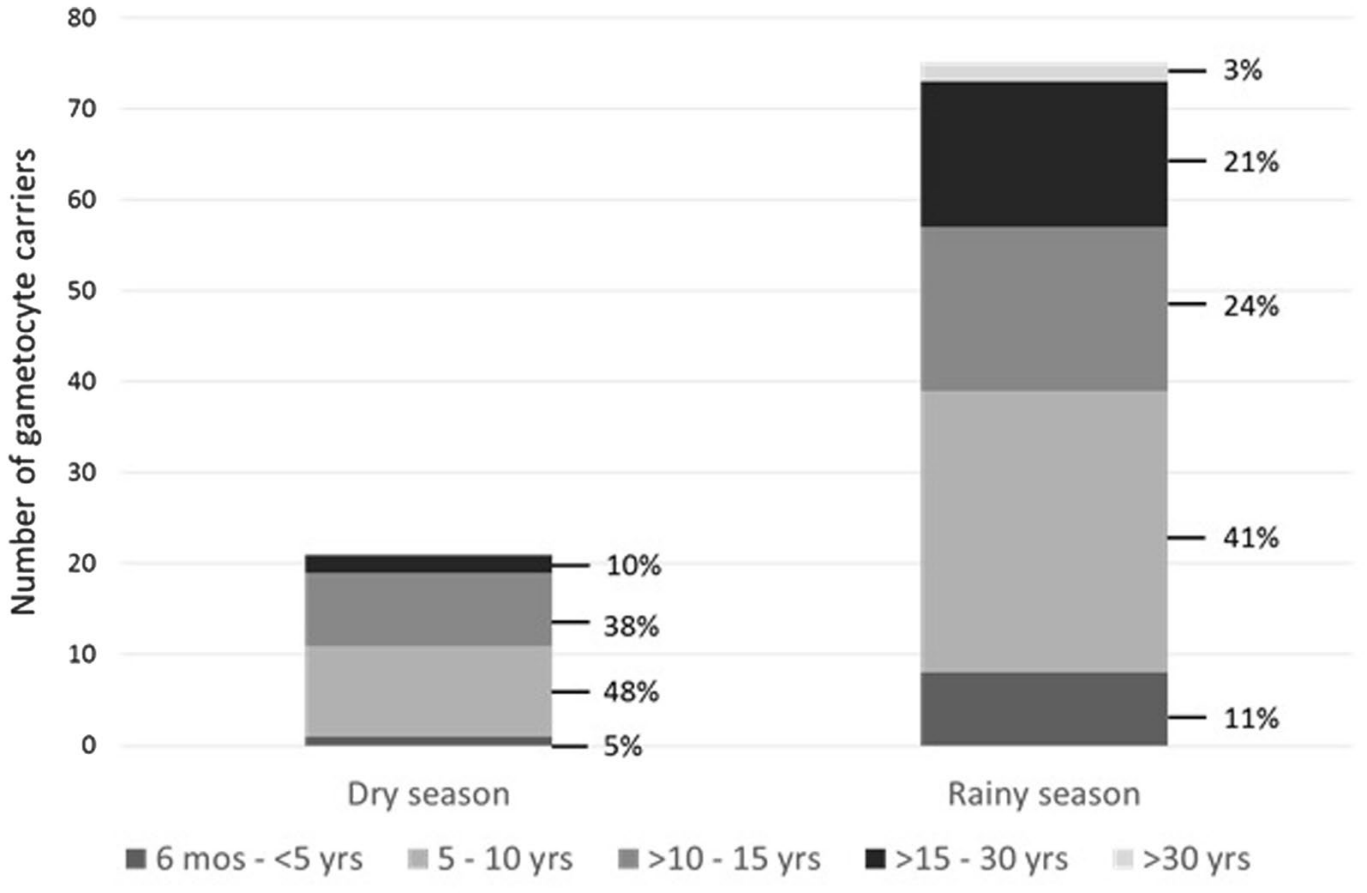
Age distribution of molecularly-detected mature gametocyte carriers by season

The strongest statistically significant predictor of gametocytes was age. Among PCR-positive *P. falciparum*-infected individuals, school-age children had 2.77 times greater odds of having gametocytes than adults (95% CI 1.47–5.19). The odds that infected young children had gametocytes were elevated compared to infected adults, but not significantly (POR 1.72, 95% CI 0.62–4.79), and less so than those of school-age children. The age distribution of identified gametocyte carriers by season further highlighted the importance of school-age children as potential transmission reservoirs, particularly during the dry season, where children aged 5–15 years old represented 85% of all gametocyte-carrying infections as detected by the molecular strategy (Fig. 5).

The power to detect other statistically significant associations was limited by few gametocytaemic individuals in certain categories. Neither EA transmission intensity nor season was significantly associated with the prevalence of gametocytes in *P. falciparum*-infected individuals. After adjusting for age, season, and EA transmission intensity, individuals who lived in more rudimentary (unfinished) houses were significantly more likely to have gametocytes when infected than those in finished houses (POR = 2.24, 95% CI 1.16–4.31). For most variables described above, the directions of the associations with gametocyte presence among PCR-positive parasitaemic individuals tended to mirror the directions of the associations with gametocyte and parasite prevalence in the total study population.

### Predictors of gametocyte density among PCR-positive infected individuals

As gametocyte density is known to be associated with infectiousness, regression models were used to assess the association of density with all potential predictors. The data were highly overdispersed, suggesting that negative binomial distributions were a better fit than Poisson. Given the large number of zero values, zero-inflated negative binomial models were compared to negative binomial models and were found to be strongly superior (Vuong test statistic, p < 0.0001) in all cases (Additional file 2). The logit component of the zero-inflated models involved the statistically significant predictors of any gametocyte carriage in PCR-positive people from Table 3–age category and household construction quality. Interestingly, the parasite density as estimated by microscopy was not found to be a significant predictor of the density of gametocytes (relative density = 1.00, 95% CI 0.997–1.01). Those factors that were significantly associated with gametocyte density in crude models (Additional file 2) were added to a final adjusted model (Table 4).

**Table 4:** Predictors of gametocyte density among those PCR-positive for *P. falciparum,* n = 219

	PCR+, n	Gametocyte carriers, n (%)	Gametocytes/μL, mean (σ)	Adjusted relative density estimate (95% CI)^a^	p value
*Season*					
Dry, 2012	52	22 (42.3%)	13.5 (306.2)	1.00 (ref)	
Rainy, 2013	167	77 (46.1%)	19.5 (1395.6)	1.13 (0.87–1.47)	0.36
*Age*					
Young children, 6 months to <5 years old	21	9 (42.9%)	21.8 (958.6)	1.03 (0.68–1.54)	0.90
School-age children, 5–15 years old	127	68 (53.5%)	18.9 (571.4)	*0.69 (0.53*–*0.89)*	0.005
Adults, ≥16 years old	71	22 (31.0%)	15.4 (2237.5)	1.00 (ref)	
*Household construction*					
Finished	76	28 (36.8%)	12.2 (317.8)	1.00 (ref)	
Unfinished	143	71 (49.7%)	21.2 (1555.4)	*1.31 (1.03*–*1.65)*	0.025
*Antimalarial in previous 2* *weeks* ^*b*^					
Yes	8	3 (37.5%)	30.4 (3156.8)	*2.46 (1.34*–*4.50)*	0.004
No	211	96 (45.5%)	17.6 (1073.0)	1.00 (ref)	
Zero-inflated binomial model characteristics					
Logit predictors: Age category, household construction					
Dispersion				0.25 (0.19–0.34)	
Vuong test Z-score (vs. negative binomial model)				6.04	<0.0001

CI Confidence interval; PCR Polymerase chain reaction

Italized values are those where the 95% CI does not contain 1.0

^a^Estimates based on zero-inflated negative binomial model with age category and household construction included in the logit portion of the model, based on finding in Table 3. Estimates are adjusted for all other factors in this table

^b^Antimalarials included were lumefantrine–artemether, chloroquine, quinine, or sulfadoxine–pyrimethamine

Although school-age children have the highest odds of carrying gametocytes when infected, their gametocyte density was 0.69 (95% CI 0.53–0.89) times that of adults and 0.67 (95% CI 0.46–0.97) times that of children under 5, controlling for season, household construction, and recent antimalarial use. There was little difference in the density comparing young children to adults (relative density = 1.03, 95% CI 0.68–1.54). Season was not a significant predictor after adjustment for the other factors, but living in unfinished households was significantly associated with 31% higher gametocyte density (95% CI 3–65%), and having recently taken an antimalarial was significantly associated with 146% higher density (95% CI 34–350%), though this estimate was based on only 8 individuals who had taken anti-malarials in the previous 2 weeks.

## Discussion

Molecular testing identified that a large proportion of study participants in southern Malawi who have *P. falciparum* infections carry mature gametocytes, even in a community-based sample with predominantly asymptomatic, often submicroscopically infected, people. These gametocyte carriers are potential contributors to ongoing transmission. About half of the patent infections had mature gametocytes by qRT-PCR, but these represented only two-thirds of all gametocytaemic individuals in the study population; one third of all gametocyte carriers in the study population had submicroscopic infections. Interestingly, the density of parasites estimated by microscopy was not significantly associated with gametocyte density estimated by qRT-PCR.

The most important individual-level predictors of gametocyte carriage in the study population were age and household construction quality. Those living in unfinished houses had higher odds of carrying gametocytes when infected than those that lived in more finished houses, and also had significantly higher gametocyte densities when infected. The associations between gametocytes and poor household construction are new findings, and suggest a possible direct causal pathway for the more indirect association between low SES and infection risks. Interventions focusing on improved household construction could potentially reduce exposure to mosquito bites and aid with interrupting transmission, but confirmation of such findings is needed, and the potential strategies for effective interventions would need to be carefully developed.

School-age children (5–15 years of age) had higher odds of being both parasitaemic and gametocytaemic in comparison to young children and adults, even after adjustment for other key predictors and after accounting for household- and EA-level clustering in the data. Among those who were PCR-positive for *P. falciparum* infection, school-age children were also the most likely to carry gametocytes, though they tended to have lower gametocyte densities than other age groups.

Most prior community-based studies that have reported gametocyte infection patterns have described a monotonic decrease in gametocyte prevalence by age rather than a peak in school-age children [11–17, 19, 20, 22–24, 28]. Only four studies, two that used microscopy [18, 26] and two that used molecular methods [21, 27] reported age-distributions similar to what we detected, with higher prevalence of and proportion of infections with gametocytes in school-age children. The present paper is the first report on the epidemiology of gametocyte infections in Malawi. Differences between the current results and those from previous studies may be attributable to the improved sensitivity of molecular assays relative to earlier studies based on microscopy or geographic differences in transmission epidemiology. Alternatively, there may have been a true shift in the age-distribution of gametocyte carriage compared to that of the population in earlier studies, possibly related to the intensive efforts to reduce malaria in children <5 years of age in the past decade. Additional prospective studies in the same geographic region, with the same detection methodology and information on antibody responses, are necessary to disentangle these complex relationships. Regardless, the current findings have important population-level implications for ongoing malaria control efforts. School-age children represent the largest group of gametocyte carriers in the study area in southern Malawi, thereby suggesting that interventions that focus solely on younger children may be unlikely to reduce and interrupt transmission. Additional studies using sensitive detection methods could evaluate whether these age distributions are consistent with what occurs in other endemic settings.

By applying the age-specific gametocyte prevalence from this study to the age structure of the Malawian population according to the most recent (2008) national census [50], an estimated 57.6% of gametocyte carriers nationwide are school-age children, 11.8% are young children, and 30.5% are adults ≥16 years of age. Furthermore, school-aged children made up 85% of all gametocyte carriers at the end of the dry season, suggesting that this demographic group may play a particularly important role in maintaining and reestablishing transmission during and after the dry season. As not all gametocyte carriers are infectious, studies that correlate gametocyte presence, especially among people whose infections are submicroscopic, with measures of infectiousness to mosquitoes are needed to fully understand the age-specific contribution to infection, particularly in light of the lower average gametocyte density found among school-age children. A recent publication by Stone et al. [51] attempted to define the proportion of new mosquito infections transmitted by age, combining such xenodiagnostic data from a number of other studies. Stone et al. estimated that school-aged children made up about 40% of the proportion of the population that was infectious, but were the source of more than half of new mosquito infections thanks to adjustments for body size and relative exposure to mosquito biting [51]. Thus, these findings support the conclusion that school-age children are critical reservoirs of infection that should be considered in interventions aimed at interruption of transmission, and suggest that this is likely consistent across other endemic settings.

The selected gametocyte marker contained exons, supporting the specificity of the results; however, screening individuals using PCR before performing qRT-PCR testing limited the sensitivity of these gametocyte data to that of PCR in the ICEMR Malawi laboratory. Our gametocyte prevalence estimates therefore represent minimum values, and do not describe gametocyte carriage among individuals whose total parasite burdens were below the limit of detection of the screening PCR. Use of ultra-sensitive PCR or testing all PCR-negative samples by qRT-PCR would likely reveal additional gametocyte carriers in the population.

There is potential for selection bias in the sampled population, as individuals who were not at home at the time of survey were not included in the blood sampling and gametocyte testing. Young children were more likely to be at home than older household members, and women more likely than men. Thus, selection bias in the associations of age and sex with gametocyte carriage could arise if the older children/adults and men who were sampled were more often present at home because they were ill with higher infection prevalences than their unsampled counterparts. However, analyses showed that recent symptom status was not associated with the odds of either gametocytes or any *P. falciparum* parasites. Thus, selection bias is unlikely to have significantly impacted the inferences.

More critically, the cross-sectional nature of the data poses a number of limitations. By identifying prevalent, but not incident infections, associations that we reported may be related to either differences in risk for developing gametocytes when infected, differences in the duration of gametocyte carriage, or a combination of the two. This distinction may be irrelevant to identifying targets for transmission interruption, but could influence which intervention types would most effectively reduce the number of humans who are acting as infectious parasite reservoirs. Longitudinal research could delineate the specific mechanisms linking age and gametocytogenesis, duration of infection, and the survival of gametocytes over the course of infections. The cross-sectional observations also precluded assessment over time, particularly patterns of seasonality and of gametocyte persistence between rainy seasons.

Gametocyte density is associated with infectiousness to mosquito vectors [10, 30], so low-density gametocyte carriers may not contribute equally to transmission as those with higher-density infections [9]. However, previous research has indicated that submicroscopic infections with gametocyte densities of <1 gametocyte/µL can potentially result in *P. falciparum* transmission to naïve vectors [10, 30, 52]. Future research could directly evaluate predictors of infectiousness using direct skin or membrane-feeding assays combined with epidemiologic data on gametocytes; such observations would help parameterize dynamic mathematical models to estimate the number of new infections caused by low density asymptomatic infections, and how this differs across different age groups.

In addition to gametocyte density, both the duration of gametocyte carriage and the frequency of biting by uninfected *Anopheles* mosquitoes influence the importance of human reservoir groups to transmission. Even if low-density, asymptomatic infections results in transmission to a smaller percentage of feeding mosquitoes than higher-density symptomatic infections, the absolute number of infected mosquitoes could be larger if more vectors feed on low-density gametocyte carriers over the course of their infections. In fact, mathematical models predict that biting heterogeneity dramatically increases the persistence of transmission compared to models that assume homogeneous biting. Increasing body size/surface area is thought to drive an increase in vector feeding with host age, such that school-age children and adults are bitten more frequently than young children [51, 53, 54]. Further, differences in bed net use contribute to differences in availability to *Anopheles* vectors. Given that school-age children in this study are the most likely to have gametocytes and have been found to be least likely to use bed nets in this area [55], they are likely to play a key role in the persistence of *P. falciparum* transmission in southern Malawi.

## Conclusions

These findings add to a small body of research that has used sensitive molecular detection methods to demonstrate age-specific epidemiology of previously undetected *P. falciparum* gametocyte infections. In these community-based cross-sectional observations, these asymptomatic infections, especially in school-age children, comprise a large proportion of the gametocyte carriers in the population. Interventions that exclude school-age children in high-transmission settings are unlikely to fully interrupt transmission of *P. falciparum* and ultimately achieve the goal of malaria elimination.

## Additional files

**Additional file 1.** Microscopy vs. molecular results for detection of gametocytes by season. Description: Table comparing microscopy results to the molecular testing results for the detection of gametocytes, with calculations of sensitivity and specificity using molecular testing at the gold standard.

**Additional file 2.** Crude estimates of relative gametocyte density among PCR + individuals, n = 219. Description: Table presenting the crude associations between potential predictors of interest and estimated gametocyte densities among individuals that were positive for *P. falciparum* infection by qPCR, comparing both negative binomial and zero-inflated negative binomial regression by the Vuong test statistic.

## Abbreviations

cDNA: complementary DNA
CI: confidence interval
EA: enumeration area
gDNA: genomic DNA
ICEMR: International Center of Excellence for Malaria Research
IRB: Institutional Review Board
IRS: indoor residual spraying
ITN: insecticide-treated net
LA: lumefantrine-artemether
PCA: principal components analysis
PCR: polymerase chain reaction
POR: prevalence odds ratio
qRT-PCR: quantitative reverse transcription polymerase chain reaction
QT-NASBA: quantitative nucleic acid sequence based amplification
REDCap: research electronic data capture
SAS: statistical analysis system
SES: socioeconomic status
SP: sulfadoxine–pyrimethamine
WBC: white blood cell
