# Supplementary material for: High prevalence of Plasmodium falciparum gametocyte infections in school-age children using molecular detection: patterns and predictors of risk from a cross-sectional study in southern Malawi
Source: Malar J. 2016 Nov 4;15:527. doi: 10.1186/s12936-016-1587-9 (PMC5096312; doi:10.1186/s12936-016-1587-9)
Supplement: Supplementary file 1 — Additional file 1. Microscopy vs. molecular results for detection of gametocytes by season. Description: Table comparing microscopy results to the molecular testing results for the detection of gametocytes, with calculations of sensitivity and specificity using molecular testing at the gold standard. [file 12936_2016_1587_MOESM1_ESM.docx]

**Additional File 1.** Microscopy vs. molecular results for detection of gametocytes by season*

| **Dry season 2012** | | | | | |
| --- | --- | --- | --- | --- | --- |
|  |  | **Microscopy results** | |  |  |
|  |  | Gametocyte + | Gametocyte - | Total |  |
| **Molecular results** | Gametocyte + | 4 | 18 | 22 | **Sensitivity: 18.2%** |
|  | Gametocyte - | 1 | 581 | 582 | **Specificity: 99.8%** |
|  | Total | 5 | 589 | 604 |  |
| **Rainy season 2013** | | | | |  |
|  |  | **Microscopy results** | |  |  |
|  |  | Gametocyte + | Gametocyte - | Total |  |
| **Molecular results** | Gametocyte + | 19 | 58 | 77 | **Sensitivity: 24.7%** |
|  | Gametocyte - | 11 | 808 | 819 | **Specificity: 98.7%** |
|  | Total | 30 | 866 | 896 |  |
| *****Note: Calculation of sensitivity and specificity were for microcopy using molecular testing as the gold standard. Molecular testing was specific to *P. falciparum*; microscopists recorded the presence of gametocytes of any *Plasmodium* species. | | | | | |
