# Supplementary material for: High prevalence of Plasmodium falciparum gametocyte infections in school-age children using molecular detection: patterns and predictors of risk from a cross-sectional study in southern Malawi
Source: Malar J. 2016 Nov 4;15:527. doi: 10.1186/s12936-016-1587-9 (PMC5096312; doi:10.1186/s12936-016-1587-9)
Supplement: Supplementary file 2 — Additional file 2. Crude estimates of relative gametocyte density among PCR + individuals, n = 219. Description: Table presenting the crude associations between potential predictors of interest and estimated gametocyte densities among individuals that were positive for P. falciparum infection by qPCR, comparing both negative binomial and zero-inflated negative binomial regression by the Vuong test statistic. [file 12936_2016_1587_MOESM2_ESM.docx]

|  | **PCR+, n** | **Gametocyte carriers,**  **n (%)** | **Gametocytes/μL, mean (σ)** | **Relative density estimate (95% CI), negative binomial model** | **Relative density estimate (95% CI), zero-inflated* negative binomial model** | **Vuong test statistic, p-value (in favor of zero-inflated model)** |
| --- | --- | --- | --- | --- | --- | --- |
| Season |  |  |  |  |  |  |
| Dry, 2012 | 52 | 22 (42.3%) | 13.5 (306.2) | 1.00 (ref) | 1.00 (ref) | 4.65, p<0.0001 |
| Rainy, 2013 | 167 | 77 (46.1%) | 19.5 (1395.6) | 1.44 (0.64 – 3.25) | **1.33 (1.01 – 1.75)** |  |
| EA transmission intensity ^α^ |  |  |  |  |  |  |
| Low | 22 | 10 (45.5%) | 23.6 (1056.8) | 1.00 (ref) | 1.00 (ref) | 4.63, p<0.0001 |
| Medium | 93 | 44 (47.3%) | 19.4 (1765.1) | 0.82 (0.24 – 2.77) | 0.79 (0.53 – 1.17) |  |
| High | 104 | 45 (43.3%) | 15.7 (608.9) | 0.67 (0.20 – 2.22) | 0.70 (0.47 – 1.04) |  |
| Parasite density ^γ^ | 215 |  | r=0.09 (p=0.17) | 1.01 (0.98 – 1.04) | 1.00 (0.997 – 1.01) | 4.47, p<0.0001 |
| Age |  |  |  |  |  |  |
| Young children, 6 mo -<5 yo | 21 | 9 (42.9%) | 21.8 (958.6) | 1.42 (0.40 – 5.07) | 1.02 (0.66 – 1.59) | 5.80, p<0.0001 |
| School-age children, 5 -15 yo | 127 | 68 (53.5%) | 18.9 (571.4) | 1.23 (0.58 – 2.63) | **0.71 (0.54 – 0.93)** |  |
| Adults, ≥16 yo | 71 | 22 (31.0%) | 15.4 (2237.5) | 1.00 (ref) | 1.00 (ref) |  |
| Household construction |  |  |  |  |  |  |
| Finished | 76 | 28 (36.8%) | 12.2 (317.8) | 1.00 (ref) | 1.00 (ref) | 4.84, p<0.0001 |
| Unfinished | 143 | 71 (49.7%) | 21.2 (1555.4) | 1.73 (0.84 – 3.58) | **1.29 (1.001 – 1.67)** |  |
| Bednet use on previous night |  |  |  |  |  |  |
| Slept under a net | 137 | 66 (48.2%) | 19.1 (1384.4) | 1.00 (ref) | 1.00 (ref) | 4.54, p<0.0001 |
| Net available but not used | 67 | 28 (41.8%) | 17.4 (827.7) | 0.91 (0.43 – 1.96) | 1.05 (0.81 – 1.37) |  |
| No nets in household | 15 | 5 (33.3%) | 11.8 (359.5) | 0.62 (0.15 – 2.51) | 0.90 (0.52 – 1.53) |  |
| SES Quartile |  |  |  |  |  |  |
| Lowest | 60 | 35 (58.3%) | 28.4 (2736.2) | 2.58 (0.66 – 10.09) | 1.47 (0.90 – 2.41) | 5.28, p<0.0001 |
| 2^nd^ | 74 | 29 (39.2%) | 15.2 (700.1) | 1.38 (0.36 – 5.25) | 1.17 (0.71 – 1.94) |  |
| 3^rd^ | 67 | 29 (43.3%) | 13.9 (339.6) | 1.27 (0.33 – 4.88) | 0.97 (0.59 – 1.60) |  |
| Highest | 18 | 6 (33.3%) | 11.0 (278.1) | 1.00 (ref) | 1.00 (ref) |  |
| Eaves |  |  |  |  |  |  |
| Closed | 150 | 65 (43.3%) | 16.9 (1281.6) | 1.00 (ref) | 1.00 (ref) | 4.51, p<0.0001 |
| Open | 69 | 34 (49.3%) | 20.7 (838.6) | 1.23 (0.58 – 2.59) | 1.08 (0.85 – 1.38) |  |
| Fever in previous 2 wks |  |  |  |  |  |  |
| Yes | 36 | 18 (50.0%) | 22.5 (1142.0) | 1.31 (0.51 – 3.32) | 1.16 (0.86 – 1.56) | 4.51, p<0.0001 |
| No | 183 | 81 (44.3%) | 17.2 (1142.3) | 1.00 (ref) | 1.00 (ref) |  |
| Antimalarial in previous 2 wks^δ^ |  |  |  |  |  |  |
| Yes | 8 | 3 (37.5%) | 30.4 (3156.8) | 1.73 (0.27 – 10.91) | **2.11 (1.11 – 4.03)** | 4.40, p<0.0001 |
| No | 211 | 96 (45.5%) | 17.6 (1073.0) | 1.00 (ref) | 1.00 (ref) |  |
| IRS in previous yr |  |  |  |  |  |  |
| Yes | 31 | 17 (54.8%) | 25.0 (856.4) | 1.45 (0.54 – 3.87) | 1.18 (0.87 – 1.60) | 4.44, p<0.0001 |
| No | 185 | 82 (44.3%) | 17.2 (1197.8) | 1.00 (ref) | 1.00 (ref) |  |
| Sex |  |  |  |  |  |  |
| Male | 91 | 44 (48.4%) | 18.0 (572.1) | 0.99 (0.49 – 2.01) | 0.88 (0.70 – 1.11) | 4.93, p<0.0001 |
| Female | 128 | 55 (43.0%) | 18.1 (1552.8) | 1.00 (ref) | 1.00 (ref) |  |
| CI Confidence interval  EA Enumeration Area  IRS Indoor residual spraying  PCR Polymerase chain reaction  SES Socioeconomic status  **Bolded values are those where the 95% CI does not contain 1.0 or the p-value is <0.05.**  * Zero-inflated negative binomial model included age category and household construction quality as the predictors in the logit portion of the  model, based on the analysis presented in Table 3.  α Tertiles of parasite prevalence established for all 30 EAs from the first survey (rainy season 2012) data were used as a proxy estimate of  transmission intensity in the EA. See Methods for details.  δ Antimalarials included were lumefantrine-artemether, chloroquine, quinine, or sulfadoxine-pyrimethamine  γ Parasite density estimated based on microscopy. The relative density estimate was reported per 1000/µL increase in parasite density | | | | | | |

**Additional File 2.** Crude estimates of relative gametocyte density among PCR+ individuals, n=219
